# Use B-factor related features for accurate classification between protein binding interfaces and crystal packing contacts

**DOI:** 10.1186/1471-2105-15-S16-S3

**Published:** 2014-12-08

**Authors:** Qian Liu, Zhenhua Li, Jinyan Li

**Affiliations:** 1 Advanced Analytics Institute and Centre for Health Technologies, Faculty of Engineering and IT, University of Technology Sydney, Broadway, NSW, 2007, Australia; 2 School of Computer Engineering, Nanyang Technological University, 50 Nanyang Ave, Singapore, 639798, Singapore

## Abstract

**Background:**

Distinction between true protein interactions and crystal packing contacts is important for structural bioinformatics studies to respond to the need of accurate classification of the rapidly increasing protein structures. There are many unannotated crystal contacts and there also exist false annotations in this rapidly expanding volume of data. Previous tools have been proposed to address this problem. However, challenging issues still remain, such as low performance when the training and test data contain mixed interfaces having diverse sizes of contact areas.

**Methods and results:**

B factor is a measure to quantify the vibrational motion of an atom, a more relevant feature than interface size to characterize protein binding. We propose to use three features related to B factor for the classification between biological interfaces and crystal packing contacts. The first feature is the sum of the normalized B factors of the interfacial atoms in the contact area, the second is the average of the interfacial B factor per residue in the chain, and the third is the average number of interfacial atoms with a negative normalized B factor per residue in the chain. We investigate the distribution properties of these basic features and a compound feature on four datasets of biological binding and crystal packing, and on a protein binding-only dataset with known binding affinity. We also compare the cross-dataset classification performance of these features with existing methods and with a widely-used and the most effective feature interface area. The results demonstrate that our features outperform the interface area approach and the existing prediction methods remarkably for many tests on all of these datasets.

**Conclusions:**

The proposed B factor related features are more effective than interface area to distinguish crystal packing from biological binding interfaces. Our computational methods have a potential for large-scale and accurate identification of biological interactions from the experimentally determined structural data stored at PDB which may have diverse interface sizes.

## Background

With the breakthrough of protein structure determination technologies, in particular X-ray crystallography, rapidly increasing 3D structures of proteins become available. For example, PDB (protein data bank) has stored 90,358 entries which are solved by X-ray crystallography as of July 2014. These quaternary structures can be used to uncover the binding mechanisms of proteins and to annotate protein functions. However, crystal packing contacts, which are a kind of false protein binding, also exist in PDB to blur the analysis of quaternary structures. In fact, crystal packing is due to the artifact of the crystallographic packing environments and it is randomly formed during the crystallization process. It does not occur in solution or in physiological states [1]. The immediate question is how to accurately determine whether a crystal contact produced from a PDB entry is a true biological interaction. This problem is difficult especially when a protein complex consists of a large number of protein chains, a common situation in PDB and also in real biological systems.

This research problem has attracted intensive interests. Methods have been proposed to understand the difference of interfacial properties between biological binding and crystal packing. For example, biological interfaces were found to be much larger [2-8], or more conserved than crystal packing [2,3], or more abundant in aromatic residues [3]. Biological interactions were also found to have different residue composition from the rest of protein surfaces [4,9,10], while crystal packing interfaces possess similar composition to the rest of protein surfaces as a whole [8].

Complicated computational methods have also been proposed to classify true biological binding and false binding. An idea is to break an interface down to contacting atomic or residue pairs, and then uses the enrichment or frequency of these pairs as features for the classification. Based on the atomic pair representation idea, Weng's group [11] and Klebe's group [12] have both utilized machine-learning algorithms to construct effective classifiers for distinguishing different types of protein binding, such as crystal packing, permanent and transient interactions [11,12]. Liu *et al*. have used a new definition of atomic contacts named *β *contacts in atomic pair representation for interfaces, and demonstrated that it is a novel idea to outperform the existing methods in distinguishing crystal packing from homodimers [13]. Using residue pairs to describe interfaces, Bernauer *et al*. have constructed an SVM classifier DiMoVo for identifying biological protein interactions [14]. Liu and Li have designed the propensity vector of residue contacts within the O-ring to develop OringPV for the distinction between crystal packing and biological interactions [15]. Many other features have also been used. For example, the PITA method scores crystal packing using the properties of contact size and chemical complementarity [16]. Zhu *et al*. [3] have extracted six properties from interfaces, such as interface size, amino acid composition and gap volume, and then used them as an SVM input to train their NOXclass classifier to discriminate between crystal packing, obligate and non-obligate interactions [3]. Recently, Capitani's group [17] have proposed to use core size and evolutionary metrics of interfacial residues to classify small biological interfaces from large crystal contacts. Their method EPPIC can outperform a widely-used method PISA [18].

Despite the intensive research on the characterization of crystal packing and biological binding, it still remains an important issue to design a good method which can be always effective across multiple datasets containing interfaces of diverse sizes, and especially on those datasets where crystal packing and biological binding have similar interface sizes [14,17]. It is even more challenging to detect one single discriminative feature which can clearly characterize crystal packing interfaces having different sizes across multiple datasets.

In this work, we propose to use B factor to distinguish biological interfaces from crystal packing contacts. B factor is a measure to capture the atomic vibrational motion. We propose to use three features derived from B factor for this classification problem. One is denoted as ΣB; it is the sum of the normalized B factors of the interfacial atoms at a binding interface. The second is the ratio of ΣB over the logarithm of *min_r _*+ 1 (the smaller one of the average numbers of residues per chain in the two units of an interaction). This feature is denoted by avgΣB. The third feature is denoted by avgNo.B which represents the ratio of the number of interfacial atoms with a negative normalized B factor over the logarithm of *min_r _*+ 1. The fourth new feature is a compound feature by integrating avgΣB and avgNo.B through multiplication to amplify these two features' collective synergy.

To show the effectiveness and the interpretability of the four features, we visualize their distribution properties from four datasets of biological binding and crystal packing, and from a biological protein-protein and protein-peptide binding dataset newly constructed from *PDBbind *[19]. For the protein interactions in this new dataset, their binding affinity is known and the complexes have diverse interface sizes.

Because interface area is considered as one of the most effective features by the existing research, we especially compare our features with interface area. To show the overall classification performance of these features, we also compare the cross-dataset classification performance of each of the four features with the performances achieved by the interface area approach and those by existing methods. The results have demonstrated that each of our four features, in particular avgΣB, avgNo.B and their multiplication, consistently outperforms the feature interface area and existing prediction methods across almost all of the datasets. These features based on B factor thus have a strong capability to distinguish true and false biological interfaces of diverse sizes for real-world applications.

## Data sets

Four datasets in the literature and a new dataset are used to investigate the four features derived from B factor.

The first dataset (*Bahadur*) contains 187 crystal packing interfaces and 122 biological homodimers [4,5]. DiMoVo was trained on this dataset [14].

The second dataset (*Ponstingl*) has 92 crystal packing interfaces and 76 homodimers [20]. This dataset was used by several existing works [11,12], including PITA [16] and PISA [18].

The third dataset (*BNCPCS*) comprises 75 obligate interactions and 106 crystal packing interfaces [3]. NOXclass was trained on this dataset.

The fourth dataset (*DC*) is composed of 82 crystal packing interfaces and 82 biological interfaces [17]. The uniqueness of this dataset is that crystal packing interfaces are larger and biological interfaces are smaller than those in the first three datasets. EPPIC was trained and optimized on this dataset [17].

A new dataset is constructed from the protein-protein binding and protein-peptide binding data stored at PDBbind [19]. All the complexes are annotated with a binding affinity extracted from PDBbind. The binding biological units in PDB structures are obtained using an automatic process according to the information provided in PDBbind. An interface is included in this dataset, if the PDB structure satisfies the following requirements. (i) The PDB structure is determined by X-ray crystallography rather than other techniques, and (ii) the resolution is better than 2.5 Å. (iii) In the PDB entry, the number of atoms should be 3+ times than the number of residues in order to remove those PDB entries with a possible error. (iv) In the complex, both of the binding partners have more than 5 residues. (v) In the interface, the number of atomic contacts from non-standard residues is less than 20% of all atomic contacts. This newly constructed dataset is composed of 799 protein-protein or protein-peptide complexes with binding affinity information. This dataset is denoted as *PDBbind*. It is a bench-marking dataset for testing algorithms on classifying biological binding interfaces of diverse area sizes.

## Methods

In this section, we describe what is B factor and how it is normalized. Then, we describe how to derive B factor related features to represent an interface. We also show how to detect the optimal distinguishability of each feature on training datasets and then test it on other datasets.

### B factor and its normalization

B factor is also known as temperature factor or Debye-Waller factor. It measures and quantities the uncertainty/mobility of an atom in dynamic protein 3D structures, namely, the displacement of the atomic positions from its mean position. B factor is an indicator of the relative vibrational motion or the disorder of an atom in protein crystal. It is calculated using $B^{i}=8\pi^{2}U_{i}^{2}$, where $U_{i}^{2}$ is the mean square displacement of atom *i*. B factor increases as $U_{i}^{2}$ increases. A low B factor implies that the atom is in the well-ordered parts of the structure, while a large B factor generally suggests a very high flexibility of this atom.

Protein flexibility is closely related to protein functions such as catalysis and allostery [21]. Deeply buried atoms in the core of the protein are usually rigid with a low B factor [22], and interfacial residues in protein binding complexes also have lower B-factors in comparison to the rest of the tertiary structural surface [23]. For different PDB structures, the distribution of B factors varies greatly. Thus, a normalized B factor is used in this work and calculated by Equation 1.

(1) $$\begin{array}{c} B_{norm}^{i}=\frac{B^{i}-\bar{\bar{B}}}{\delta_{B}}\times \frac{1}{1.645}\\ {\ddot{B}}_{norm}^{i}=min\left[max\left(B_{norm}^{i},-1\right),1\right] \end{array}$$

where *B^i ^*is the B factor of atom *i*, $\bar{B}$ and *δ_B _*are the mean and the standard deviation of the B factor of all atoms within a binding unit of the PDB biological complexes, and $B_{norm}^{i}$ is the normalized B factor of atom *i*. The number 1.645 is a typical threshold under a standard normal distribution, indicating the 0.05 probability of a value outside [−1.645, 1.645] for each of the two tails. *min *means the minimum of two values, while *max *returns the maximum. The first equation in Equation 1 is used to normalize and scale the 90% confidence interval of the B factor to [-1, 1]. The second equation in Equation 1 is used to set any value outside the 90% confidence interval to either -1 or 1, whichever is closer. The normalization is performed individually on each contact partner in a complex, no matter the contact is false or true.

### Using B factor related features to characterize an interface

#### Interfacial atoms

An atom from a biological unit is defined as an interfacial atom if it has at least one *β *contacts with the partner biological unit. We note that a biological unit may contain more than one chain. *β *contact is a new definition of atomic contact [13]. It requires that there is no other atom interrupting the contact. Formally, given a quaternary structure of a protein complex *p*, a *β *contact between two atoms *i *and *j *in *p *requires that (i) the spatial distance between *i *and *j *is less than a threshold *Td *plus the sum of their van der Waals radii defined by [24], (ii) *i *and *j *share a Voronoi facet in *p*'s Voronoi diagram, and (iii) the contact cannot break *p*'s *β*-skeleton. The *β*-skeleton [25] of a discrete set *p *is an undirected graph in computational geometry. In this graph, two points *i *and *j *have an edge if angle *ikj *is sharper than a threshold determined by *β*, ∀*k *∈ *p*, *k *≠ *i*, *j*. This angle threshold is denoted as ∠*β*, which actually defines a forbidden region *fr *of the contact between *i *and *j*. The forbidden region *fr *of a *β *contact usually does not cover any other atoms. Otherwise, if there is an atom *k *in *fr*, the contact between *i *and *j *is not a *β *contact. A *β *contact suggests that its two atoms should have enough direct contact area to form an important interaction. The number of atomic *β *contacts in protein binding interfaces is only a small fraction number of distance-based contacts or less than half the number of contacts in the Voronoi diagrams [13]. Interestingly, it has been demonstrated that the use of *β *contacts can achieve better prediction performance for distinguishing false binding of crystal packing from homodimers [13], for predicting binding hot spots and the change of binding free energy after mutations [26], and for estimating protein-ligand binding affinity [27].

In this work, an interfacial atom is used for further analysis if and only when the number of its local contacts across the interface is more than 2. The local contacts of an atom include the contacts of the atom itself and the contacts of its covalently-bonded nearby atoms. The covalently-bonded nearby atoms of a given atom *i *are those atoms within two covalent-bond steps from *i*. For example, given a chain of covalent bonds *i *− *j *− *k *− *l *− *m*, where − indicates a covalent bond. From *i*, the covalently-bonded step is 0 to *i*, is 1 to *j*, is 2 to *k*, is 3 to *l*, and is 4 to *m*. Thus, *i*, *j *and *k *are the covalently-bonded nearby atoms of atom *i*, while *l *and *m *are not. The requirement of the number of local contacts is used to detect non-isolated atomic contacts.

#### Four interfacial features related to B factor

**B factor score (denoted by **Σ**B) **The first feature to describe an interface is the sum of the normalized B factors of all of the interfacial atoms. That is, $\Sigma B=\sum_{j=1}^{N}{\ddot{B}}_{norm}^{i_{j}}$, where *N *is the number of interfacial atoms and *ij *is an interfacial atom, 1 ≤ *j *≤ *N*.

**Average **Σ**B (denoted by avg**Σ**B) **A recent published work has suggested that the area size of protein interfaces is related to the size of proteins [28]. Thus, we calculate the ratio of ΣB over the logarithm of *min_r _*+ 1, and name this ratio average ΣB, denoted by avgΣB. Formally, avgΣB = ΣB/*log*(*min_r _*+ 1). Here, *min_r_*is the smaller number of the average numbers of residues per chain for the two biological units in a complex. The logarithm is used to decrease the effect of *min_r _*on avgΣB when *min_r_* is extremely large.

**The number of interfacial atoms with a negative normalized B factor (denoted by No.B) **We also calculate the number of interfacial atoms having a normalized B factor less than 0. It is denoted by No.B. Similarly, we produce the ratio of No.B over *log*(*min_r _*+ 1) based on the same reason for avgΣB. This ratio feature is denoted by avgNo.B.

**A combined feature--avg**ΣB*avgNo.B We also multiply avgNo.B and avgΣB/100 as a feature to describe an interface. This feature is denoted by avgΣB*avgNo.B. The intuition behind this new feature is to amplify the collective synergy of avgΣB and avgNo.B through multiplication.

### Interface area (ΔASA)

An effective feature widely used by the existing works to distinguish biological binding and crystal packing is interface area (ΔASA). Interface area measures half of the change of a surface area upon protein complex formation. The classification performance of this feature is considered as a baseline performance here. ΔASA of a protein complex is calculated through Equation 2.

(2) $$\Delta ASA=\left(ASA_{1}+ASA_{2}-ASA_{C}\right)/2$$

where *ASA*_1 _and *ASA*_2 _are the surface areas of the two biological units of the protein complex and *ASA_C _*is the surface area of the protein complex.

Similarly, the ratio of ΔASA over the logarithm of *min_r _*+ 1 is denoted by avgΔASA. Both ΔASA and avgΔASA are compared with the B factor based features for identifying biological binding interfaces from PDB structure data.

### Optimization of the scoring threshold for each feature

For each of the features introduced above, we use the following process to find the best threshold point on a learning dataset for the classification of test data. We explore all possible split points for a feature, and assess the MCC performance with regard to every split point. Then, we collect all those split points which produce the top 10% performance, and take the average of these split points as the optimal split threshold for the feature in the learning process. This threshold is used to predict interaction types (biological binding or crystal packing) for the structure data from the other datasets. Using the average of the top 10% best split points instead of the best split point is for the purpose of increasing performance stability and generalizability of the feature. When the *PDBbind *dataset is used for learning, the value at the first 25% quantile, which is close to 0, is used as the threshold and tested on the other datasets. This is because *PDBbind *is constructed using an automatic process without manual checking, and it is possible that some true complexes are wrongly collected. The threshold value 25% is not optimal. There is no gold standard to select an optimal threshold on *PDBbind*, because only positive samples are given.

### Assessment measures

Prediction performance is measured by *precision*(*pre*.), *recall*(*rec*.), *specificity*(*spec*.) *accuracy*(*acc*.) and *MCC *whose definitions are given in Equation 3.

(3) $$\begin{array}{ccc} precision\left(pre.\right) & = & \frac{TP}{TP+FP}\\ recall\left(rec.\right) & = & \frac{TP}{TP+FN}\\ specificity\left(spec.\right) & = & \frac{TN}{TN+FP}\\ accuracy\left(acc.\right) & = & \frac{TP+TN}{TP+TN+FP+FN}\\ MCC & = & \frac{TP*TN-FP*FN}{\sqrt{\left(TP+FP\right)\left(TP+FN\right)\left(TN+FP\right)\left(TN+FN\right)}} \end{array}$$

where binding complexes are considered as the true cases, while crystal packing as the false cases; TP, FP, TN and FN are the number of true positives, false positives, true negatives and false negatives, respectively. Hence, *precision *is the number of correct binding complex predictions divided by the number of positive predictions, *recall *is the fraction of correct binding complex predictions over all true binding complexes, while *accuracy *is the number of correct predictions divided by the number of all true or false complexes.

## Results

We report cross-dataset classification performances achieved by each of the B-factor based features in comparison with the performance by the feature interface size (ΔASA). It is observed that avgΣB, avgNo.B and avgΣB*avgNo.B have much better performance than ΔASA. We then present a detailed distribution analysis for these features' scores of the protein structures from the five datasets. We also compare avgΣB with two published methods EPPIC [17] and PISA [18] to highlight our better classification performance.

### Cross-dataset classification performance by single features

Comparison between ΣB and ΔASA: ΔASA is a geometrical feature widely used by existing methods, and it is considered as an effective approach to the classification between crystal packing and true biological binding. It has been suggested to use 856 Å^2 ^[20] as a threshold to distinguish crystal packing contacts from homodimers, achieving an accuracy of 85% on the *Ponstingl *data set. In [3], it is shown that a cutoff of ΔASA at 650Å^2 ^has approximately 7% error rates on the *BNCPCS *dataset including 62 non-obligate interactions. However, these methods have limits to achieve good performance when the biological binding interfaces and crystal packing contact areas have diverse interface sizes.

Table 1 shows the classification performance for ΣB and ΔASA on the five datasets. It can be seen that ΣB has much better classification performances than ΔASA under almost all of these tests. In particular, when tested on *DC*, ΔASA has three negative MCC performance and another two low MCC values less than 0.3. But, ΣB always has positive MCC values larger than 0.3. This performance difference is mainly attributed to the hard case that similar sizes of the interface areas exist between the crystal packing contacts and the real biological binding interfaces in *DC*. Under this situation, the classification capability of ΔASA is lost.

**Table 1:** Cross-dataset classification performances.

Training dataset	Feature	Tested datasets			
		
		*BNCPCS*	*DC*	*Bahadur*	*Ponstingl*
*BNCPCS*	ΣB	*0.93(0.97)*	**0.32(0.65)**	**0.65(0.82)**	**0.82(0.91)**
	ΔASA	*0.92(0.96)*	-0.18(0.47)	0.59(0.78)	0.73(0.86)
	
	avgΣB	*0.92(0.96)*	**0.37(0.68)**	0.64(0.82)	0.80(0.90)
	avgNo.B	*0.95(0.98)*	0.25(0.60)	0.70(0.84)	**0.84(0.92)**
	avgΣB*avgNo.B	*0.94(0.97)*	0.33(0.66)	**0.70(0.85)**	0.82(0.91)
	avgΔASA	*0.91(0.96)*	-0.16(0.48)	0.64(0.81)	0.72(0.86)

*DC*	ΣB	**0.85(0.92)**	*0.38(0.69)*	**0.68(0.85)**	**0.81(0.90)**
	ΔASA	0.73(0.86)	*0.15(0.57)*	0.66(0.84)	0.62(0.80)
	
	avgΣB	**0.88(0.94)**	*0.45(0.73)*	0.73(0.87)	0.80(0.90)
	avgNo.B	0.80(0.90)	*0.46(0.72)*	0.74(0.87)	0.70(0.84)
	avgΣB*avgNo.B	0.86(0.93)	*0.45(0.73)*	**0.75(0.88)**	**0.81(0.90)**
	avgΔASA	0.76(0.88)	*0.27(0.63)*	0.68(0.85)	0.66(0.82)

*Bahadur*	ΣB	**0.84(0.92)**	**0.38(0.69)**	*0.71(0.86)*	**0.79(0.89)**
	ΔASA	0.73(0.86)	0.15(0.57)	*0.66(0.84)*	0.62(0.80)
	
	avgΣB	0.84(0.92)	0.41(0.70)	*0.75(0.88)*	0.81(0.90)
	avgNo.B	0.86(0.93)	0.33(0.66)	*0.75(0.88)*	0.77(0.88)
	avgΣB*avgNo.B	**0.88(0.94)**	**0.45(0.73)**	*0.77(0.89)*	**0.83(0.91)**
	avgΔASA	0.81(0.90)	0.21(0.60)	*0.69(0.85)*	0.69(0.84)

*Ponstingl*	ΣB	0.88(0.94)	**0.39(0.70)**	**0.69(0.85)**	*0.81(0.90)*
	ΔASA	**0.91(0.96)**	-0.18(0.47)	0.59(0.79)	*0.72(0.86)*
	
	avgΣB	0.90(0.95)	**0.43(0.71)**	0.73(0.87)	*0.82(0.91)*
	avgNo.B	**0.95(0.98)**	0.25(0.60)	0.70(0.84)	*0.84(0.92)*
	avgΣB*avgNo.B	0.90(0.95)	0.40(0.70)	**0.75(0.88)**	*0.83(0.92)*
	avgΔASA	0.92(0.96)	-0.19(0.46)	0.65(0.82)	*0.78(0.89)*

*PDBbind*	ΣB	**0.93(0.97)**	**0.38(0.68)**	**0.62(0.79)**	**0.72(0.86)**
	ΔASA	0.88(0.94)	-0.16(0.48)	0.49(0.68)	0.62(0.79)
	
	avgΣB	0.88(0.94)	**0.41(0.71)**	0.71(0.86)	0.83(0.92)
	avgNo.B	**0.92(0.96)**	0.38(0.68)	0.74(0.88)	0.80(0.90)
	avgΣB*avgNo.B	0.90(0.95)	0.38(0.69)	**0.76(0.88)**	**0.86(0.93)**
	avgΔASA	0.88(0.94)	0.02(0.51)	0.66(0.84)	0.70(0.85)

X.XX(Y.YY) represent the classification performances where X.XX is the MCC score and Y.YY is the accuracy score. The *italic numbers *are the learning performances, and thus they are not used in the comparison. The **bold-font **numbers are the better performances when comparing ΣB and avgΣB*avgNo.B with ΔASA, and ΣB with ΔASA.

When tested on the *Bahadur *and *Ponstingl *datasets, ΣB outperforms ΔASA for all cases, achieving at least 0.1 MCC improvement in 5 of the 8 cross-dataset comparisons, and achieving 0.05 - 0.1 MCC improvement in another 2 comparisons. When tested on *BNCPCS*, ΣB has also achieved higher MCC performance than ΔASA when both ΣB and ΔASA are optimized on *DC *and *Bahadur*. ΔASA has only achieved a higher MCC performance than ΣB on *BNCPCS*, when optimized on the *Ponstingl *dataset. We note that crystal packing contacts from *BNCPCS *are easy to be distinguished--both ΣB and ΔASA have achieved an accuracy higher than 0.94. When *PDBbind *is used in learning process and the other datasets are used for testing, ΣB always outperforms ΔASA remarkably.

Comparison between avgΣB and avgΔASA: When the two average-smoothed features, i.e., avgΣB and avgΔASA, are used in the classification, their performance is better than the non-smoothed features ΣB and ΔASA, respectively. This affirms that taking average is a good way to deal with the issue of relative size of an interface compared to its chains. This idea is especially meaningful when protein-peptide binding interfaces are considered for classification where peptides are usually of small sizes and the corresponding binding interfaces are always much smaller than protein-protein binding interfaces. Table 1 also shows the superior performance of avgΣB in comparison with avgΔASA for almost all of the cross-dataset tests.

The performance of avgNo.B and of avgΣB*avgNo.B: The feature avgNo.B is also useful to classify crystal packing from biological binding. But its performance is a bit unstable in comparison with ΣB or avgΣB. Nevertheless, it still has a stabler than ΔASA. The cross-dataset classification performance by avgΣB*avgNo.B (the multiplication of avgΣB and avgNo.B) is presented in the middle row of Table 1 for each of the datasets. This performance is competitive to the best performance achieved by avgΣB or avgNo.B. This feature also outperforms ΔASA and avgΔASA for almost all of the across-dataset tests.

### The value distributions of our B factor based features and the value distribution of the feature interface size

**Figure 1:**
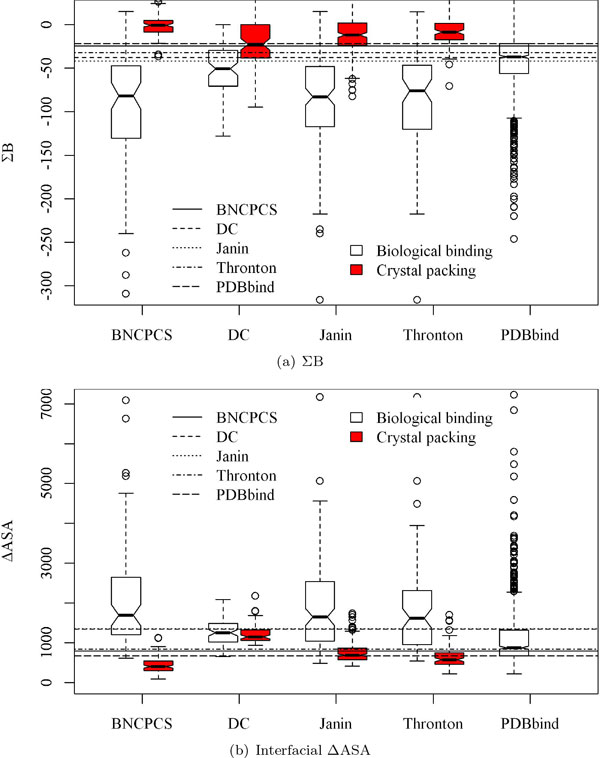
**The score distributions of ΣB and ΔASA in boxplot for the five datasets**. The p-values are shown in Table 2. The horizontal lines represent the best split points for each of the four datasets and the 25% quantile point for *PDBbind*.

**Figure 2:**
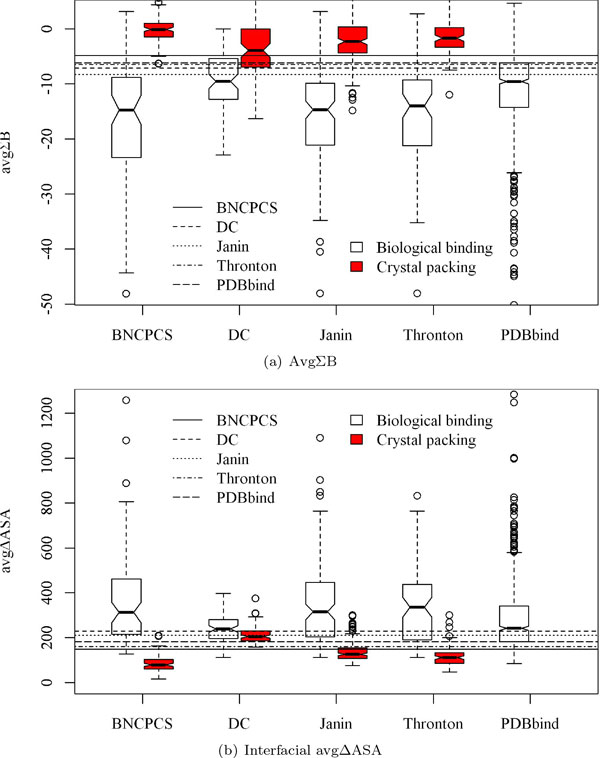
**The score distributions of AvgΣB and avgΔASA in boxplot for the five datasets**. The p-values are shown in Table 2. The horizontal lines represent the best split points for each of the four datasets and the 25% quantile point on *PDBbind*.

**Figure 3:**
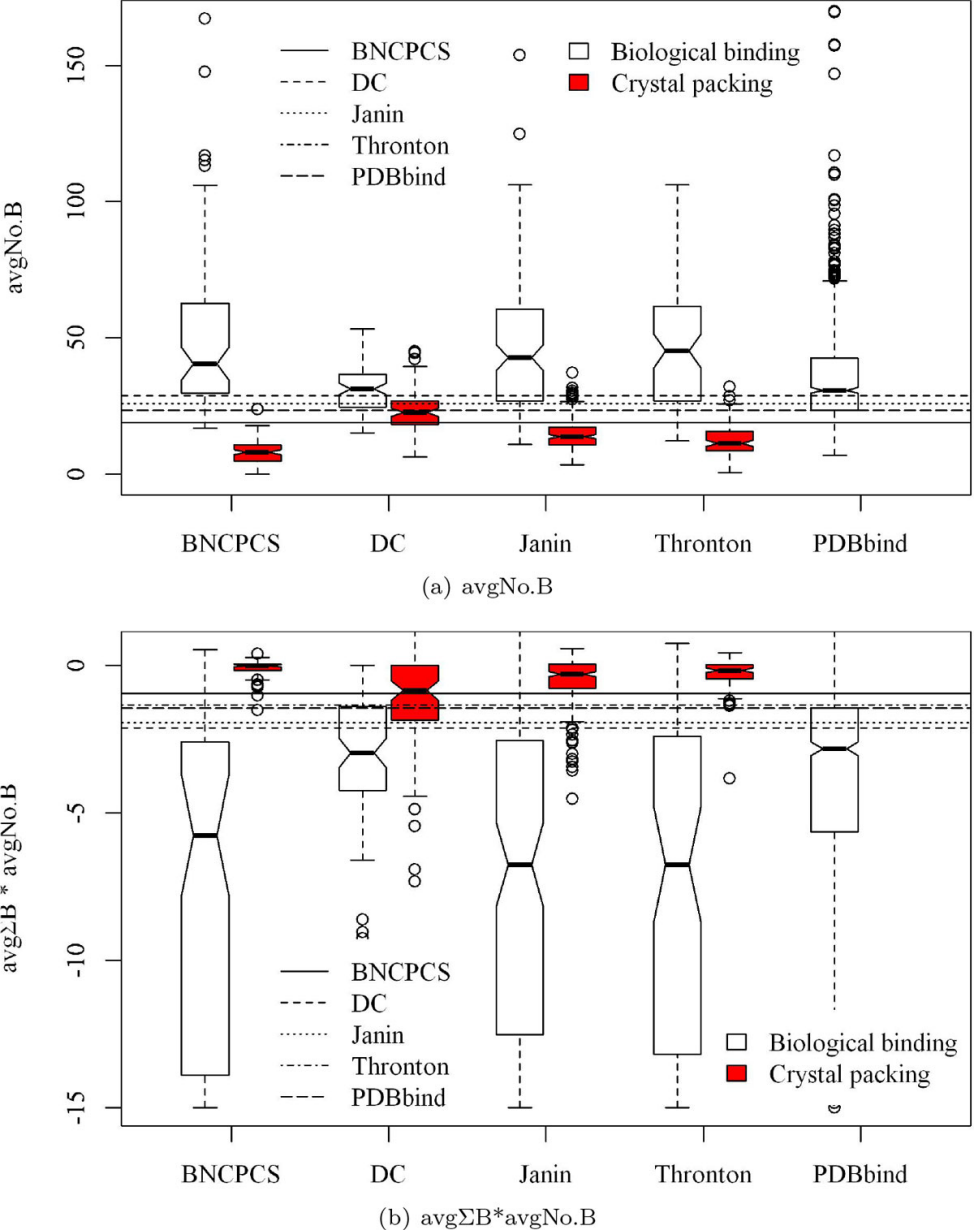
The score distributions of Average No.B (avgNo.B) and avgΣB*avgNo.B in boxplot for the five datasets. The p-values are shown in Table 2. The horizontal lines represent the best split points for each of the four datasets and the 25% quantile point on *PDBbind*. avgΣB*avgNo.B is divided by 100 for better visualization but without changing its value distribution between the two types of interfaces.

The value distributions of the features on the five datasets are drawn in Figures 1, 2 and 3. The p-values of these distributions for the two types of interfaces are reported in Table 2. It is clear from Figure 1(a) and Figure 2(a) that B factor related features such as ΣB are more powerful than interface size to distinguish between biological binding interfaces and crystal packing interfaces.

**Table 2:** p-values of different features for the two types of interfaces in the four datasets.

Feature	Datasets			
	
	*BNCPCS*	*DC*	*Bahadur*	*Ponstingl*
ΣB	9.89e-20	4.47e-09	5.68e-28	1.68e-19
ΔASA	5.58e-17	0.184	1.21e-21	4.02e-14

avgΣB	1.72e-21	4.61e-10	2.70e-31	3.02e-22
avgNo.B	2.41e-19	1.71e-09	2.15e-27	6.01e-19
avgΣB*avgNo.B	6.91e-19	6.51e-09	3.40e-28	3.07e-18
avgΔASA	4.62e-18	0.00141	2.30e-24	1.12e-16

In particular on the *DC *dataset, crystal packing contacts have very similar area sizes with those of the biological binding interfaces. Features ΣB and avgΣB can classify these two types of interfaces very well. This classification is quantified as in Table 2 where B factor related features always have much smaller and more significant p-values than those of ΔASA. However, ΔASA even has insignificant p-value 0.184 on the *DC *dataset. Features avgNo.B and avgΣB*avgNo.B (Figure 3) can also separate the two types of interfaces with a clearer boundary than ΔASA does (Figure 1(b) and Figure 2(b)).

**Figure 4:**
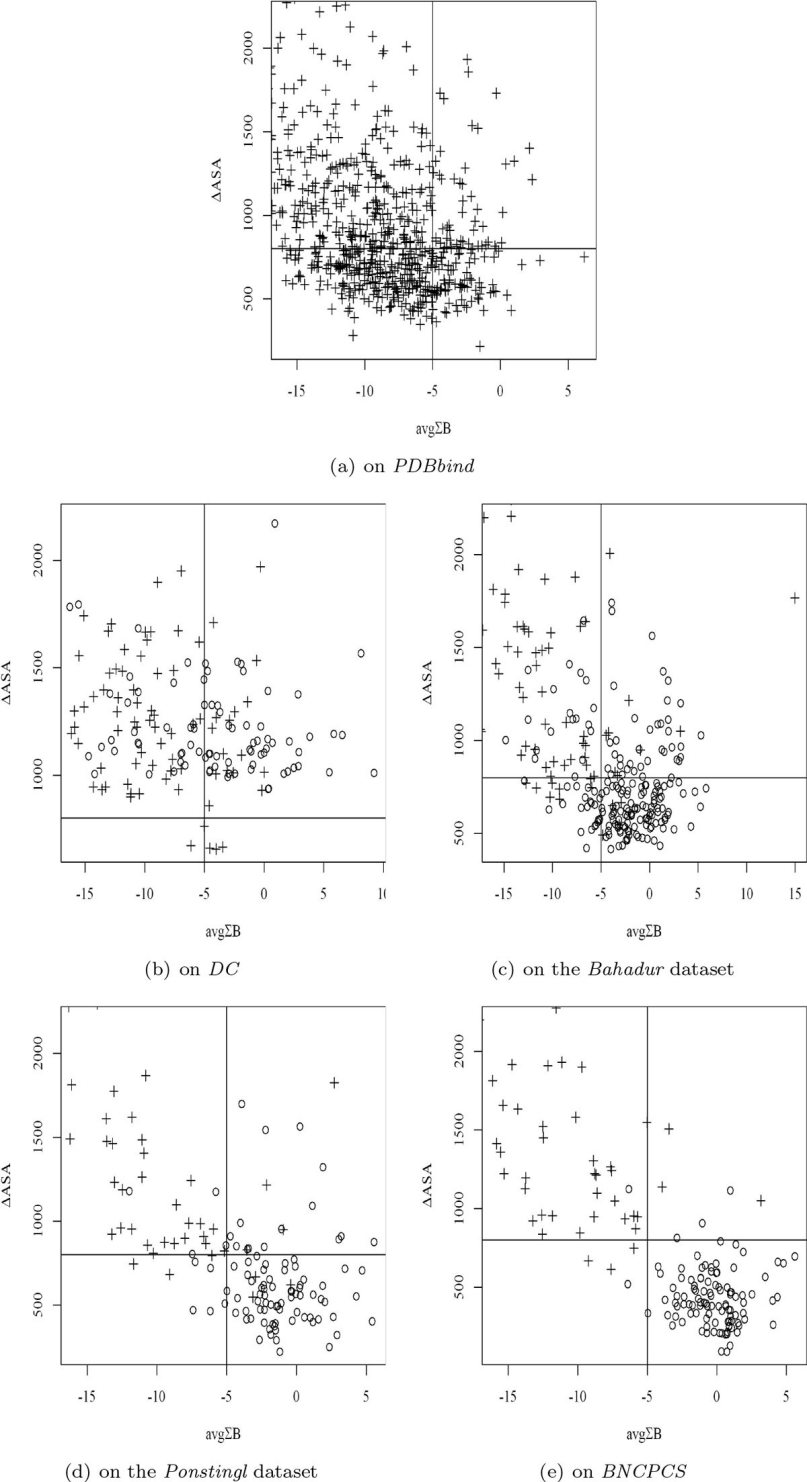
**The relationship of ΔASA and avgΣB**. Sign + represents a true binding interface, while ○ represents a crystal packing interface. Those complexes with ΔASA larger than 2200 Å or avgΣB smaller than -16 are all true binding and thus are not drawn. The horizontal lines have ΔASA = 800 Å, while the vertical lines have avgΣB = -5. Both the values are not optimized but used only for a better visualization of the different distribution across datasets.

The scatter plots of avgΣB and ΔASA on the five datasets are presented in Figure 4. Figure 4(a) indicates that ΔASA wrongly classifies many of those protein binding interfaces of *PDBbind *below the horizontal line as crystal packing contacts, while avgΣB misclassifies much less number of protein binding interfaces on the right-hand side of the vertical line (142 vs 322). Further, Figure 4(b) suggests that a cross-dataset ΔASA threshold is useless on *DC*. Figure 4(c) on the *Bahadur *dataset and Figure 4(d) on the *Ponstingl *dataset both demonstrate that many of the crystal packing contacts with a large interfaces can have a small avgΣB values and thus they can be correctly classified by avgΣB. In Figure 4(e) on *BNCPCS*, both ΔASA and avgΣB are powerful to distinguish between crystal packing and biological binding.

In conclusion, avgΣB and avgΣB*avgNo.B have a consistent classification performance across the datasets with diverse interface sizes, including those large interfaces of crystal packing and small interfaces of biological binding.

### Classification performance comparison with PISA and EPPIC

The performances by avgΣB and avgΣB*avgNo.B are compared with a widely-used method PISA and a newly published method EPPIC (Table 3). Although much less number of features are used by our approach, our single feature avgΣB can outperform both EPPIC and PISA. On both the *Ponstingl *and the *Bahadur *datasets, the MCC scores of EPPIC are quite close to those of avgΣB, and also much higher than those of PISA.

**Table 3:** Comparison with existing methods PISA and EPPIC.

Tested on	Methods	Prec	Sens	Spec	Acc	MCC
*BNCPCS*	EPPIC-core	0.98	0.76	0.99	0.90	0.79
	AvgΣB	1.00	0.85	1.00	0.94	0.88
	avgΣB*avgNo.B	1.00	0.83	1.00	0.93	0.86

*Ponstingl*	EPPIC-core	0.90	0.75	0.93	0.85	0.70
	AvgΣB	0.94	0.83	0.96	0.90	0.80
	avgΣB*avgNo.B	0.98	0.79	0.99	0.90	0.81
	EPPIC	0.92	0.90	0.87	0.89	0.76
	PISA	0.87	0.89	0.77	0.84	0.66

*Bahadur*	EPPIC-core	0.92	0.80	0.95	0.89	0.77
	AvgΣB	0.85	0.81	0.91	0.87	0.73
	avgΣB*avgNo.B	0.89	0.80	0.94	0.88	0.75
	EPPIC	0.78	0.89	0.84	0.86	0.72
	PISA	0.65	0.89	0.69	0.77	0.57

All of these methods are optimized on the *DC *dataset. EPPIC-core is the classifier using the number of core residues in interfaces according to the definition in EPPIC. The performance of EPPIC or PISA is borrowed from [17]. The scores of EPPIC and PISA are absent on the *BNCPCS *dataset.

Our method also has much higher specificity and higher precision, indicating that the predicted biological binding interfaces are more likely to be true binding. It is thus quite useful to automatically compile protein-binding datasets from PDB for large-scale structural analysis where crystal packing contacts should be correctly labeled and then excluded to enhance the analysis results.

### Feature avg ΣB can be used to correct errors in biological binding annotation: an example

**Figure 5:**
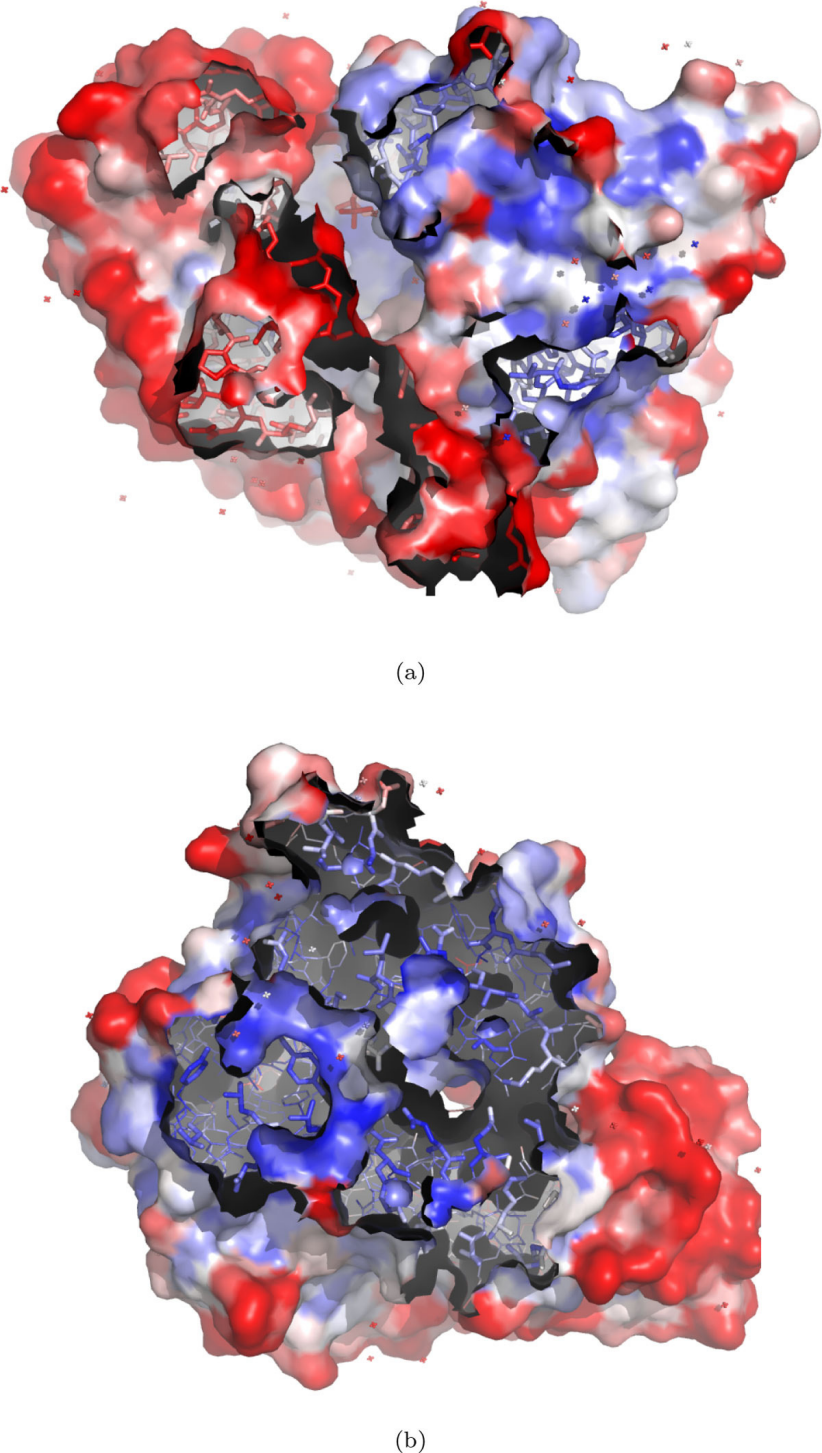
**Two interfaces derived from the PDB entry **1UBY. (a) The used dimer structure in the *Bahadur *dataset which is derived by a computational tool with regard to the biomolecule 2 of REMARK 350 in 1UBY; (b) the dimer structure determined by the authors of 1UBY. The original B factors in 1UBY are ranged between 13.22 and 83.45 according to Equation 1. The colors from blue to white and to red indicate B factors from small to large. The structures are shown in the surface view. The regions without any surface view are the binding sites on chain A. The binding sites on chain B are not shown due to the symmetry of the interfaces.

The B factor feature avgΣB is able to correct annotation errors. We demonstrate such corrections in Figure 5 by examining two derived protein complexes from PDB entry 1UBY.

Figure 5(a) shows a one-side binding site of the interface for a derived complex with regard to the biomolecule 2 of the REMARK 350 of 1UBY. This interface has a ΔASA = 1766.75 Å^2 ^and it is predicted to be dimeric by a computational tool [29]. However, there are no biological evidences so far to claim it as a true dimer. Figure 5(b) displays a one-side binding site of another derived dimeric interface (according to the biomolecule 1 of the REMARK 350 in 1UBY). This binding interface is actually recommended by the authors of 1UBY[30].

The interface in Figure 5(a) is manually mistaken as a biological binding interface in the *Bahadur *dataset. But, it is the interface in Figure 5(b), instead of that in Figure 5(a), that should be in this dataset. Feature avgΣB can correct this mistake with two reasonable evidences as follows. Firstly, the interface in Figure 5(a) has an avgΣB value of 14.96, which is in the top-right region of Figure 4(c) with '+'. This avgΣB value is extremely different from the avgΣB values of other biological binding interfaces as shown in Figure 4. Secondly, the interface in Figure 5(a) has atoms with larger B factor in red, while the interface in Figure 5(b) has atoms with much smaller B factor in blue. Thus, avgΣB can make a reasonable prediction that the interface in Figure 5(b) is dimeric and the interface in Figure 5(a) should not be. This is also consistent with the biological evidence in the REMARK 350 of 1UBY[30]. Thus, the interface in Figure 5(a) needs more biological evidences to be claimed as a true dimer. This example illustrates that the B factor feature avgΣB can be used to correct wrong annotations of biological binding interfaces.

## Conclusion

In this work, we have proposed to use B factor as a new characteristic to distinguish between crystal packing contacts and biological binding interfaces. Assessed on five datasets, all of the B factor related features have exhibited their excellent capability for classifying various biological binding interfaces with diverse interface sizes. Our B factor features have also achieved better classification performances than the widely-used feature interface size and two published methods PISA and EPPIC. In particular, the average sum of normalized B factor of interfacial atoms is a clear indictor for biological binding. As a future work, the B factor related features and our method will be employed for a large scale annotation of potential biological binding interfaces for PDB.

## Competing interests

The authors declare that they have no competing interests.

## Authors' contributions

QL designed the methods and performed the experiments. JL supervised the study. JL and ZL participated in the analysis. QL drafted the manuscript. QL, ZL and JL read and revised the manuscript. All authors approved the final version.

## Declarations

The publication charges this article were funded by an ARC Discovery Project (DP130102124).

This article has been published as part of *BMC Bioinformatics *Volume 15 Supplement 16, 2014: Thirteenth International Conference on Bioinformatics (InCoB2014): Bioinformatics. The full contents of the supplement are available online at http://www.biomedcentral.com/bmcbioinformatics/supplements/15/S16.

## References

[B1] Tuncbag N Kar G Keskin O Gursoy A Nussinov R A survey of available tools and web servers for analysis of protein-protein interactions and interfaces Brief Bioinform 2009 10 3 217 232 19240123 10.1093/bib/bbp001 PMC2671387

[B2] Valdar WSJ Thornton JM Conservation helps to identify biologically relevant crystal contacts J Mol Biol 2001 313 2 399 416 10.1006/jmbi.2001.5034 11800565

[B3] Zhu H Domingues FS Sommer I Lengauer T NOXclass: prediction of protein-protein interaction types BMC Bioinformatics 2006 7 27 10.1186/1471-2105-7-27 16423290 PMC1386716

[B4] Bahadur RP Chakrabarti P Rodier F Janin J A dissection of specific and non-specific protein-protein interfaces J Mol Biol 2004 336 4 943 955 10.1016/j.jmb.2003.12.073 15095871

[B5] Bahadur RP Chakrabarti P Rodier F Janin J Dissecting subunit interfaces in homodimeric proteins Proteins 2003 53 3 708 719 10.1002/prot.10461 14579361

[B6] Janin J Rodier F Protein-protein interaction at crystal contacts Proteins 1995 23 4 580 587 10.1002/prot.340230413 8749854

[B7] Janin J Specific versus non-specific contacts in protein crystals Nature Structural Biology 1997 4 973 974 10.1038/nsb1297-973 9406542

[B8] Carugo O Argos P Protein-protein crystal-packing contacts Protein science 1997 6 10 2261 2263 9336849 10.1002/pro.5560061021 PMC2143556

[B9] Jones S Thornton JM Analysis of protein-protein interaction sites using surface patches J Mol Biol 1997 272 121 132 10.1006/jmbi.1997.1234 9299342

[B10] Lo Conte L Chothia C Janin J The atomic structure of protein-protein recognition sites J Mol Biol 1999 285 5 2177 2198 10.1006/jmbi.1998.2439 9925793

[B11] Mintseris J Weng Z Atomic contact vectors in protein-protein recognition Proteins 2003 53 3 629 639 10.1002/prot.10432 14579354

[B12] Block P Paern J Hullermeier E Sanschagrin P Sotriffer CA Klebe G Physicochemical descriptors to discriminate protein-protein interactions in permanent and transient complexes selected by means of machine learning algorithms Proteins 2006 65 3 607 622 10.1002/prot.21104 16955490

[B13] Liu Q Kwoh CK Hoi SCH Beta Atomic Contacts: Identifying Critical Specific Contacts in Protein Binding Interfaces PLoS ONE 2013 8 4 e59737 10.1371/journal.pone.0059737 23630569 PMC3632532

[B14] Bernauer J Bahadur RPP Rodier F Janin J Poupon A DiMoVo: a Voronoi tessellation-based method for discriminating crystallographic and biological protein-protein interactions Bioinformatics 2008 24 652 658 10.1093/bioinformatics/btn022 18204058

[B15] Liu Q Li J Propensity vectors of low-ASA residue pairs in the distinction of protein interactions Proteins 2010 78 589 602 19768686 10.1002/prot.22583

[B16] Ponstingl H Kabir T Thornton JM Automatic inference of protein quaternary structure from crystals Journal of Applied Crystallography 2003 36 5 1116 1122 10.1107/S0021889803012421

[B17] Duarte J Srebniak A Scharer M Capitani G Protein interface classification by evolutionary analysis BMC Bioinformatics 2012 13 334 10.1186/1471-2105-13-334 23259833 PMC3556496

[B18] Krissinel E Henrick K Inference of Macromolecular Assemblies from Crystalline State J Mol Biol 2007 372 3 774 797 10.1016/j.jmb.2007.05.022 17681537

[B19] Cheng T Li X Li Y Liu Z Wang R Comparative Assessment of Scoring Functions on a Diverse Test Set J Chem Inf Model 2009 49 4 1079 1093 10.1021/ci9000053 19358517

[B20] Ponstingl H Henrick K Thornton JM Discriminating between homodimeric and monomeric proteins in the crystalline state Proteins 2000 41 47 57 10.1002/1097-0134(20001001)41:1<47::AID-PROT80>3.0.CO;2-8 10944393

[B21] Yuan Z Bailey TL Teasdale RD Prediction of protein B-factor profiles Proteins: Struct, Funct, Bioinf 2005 58 4 905 912 10.1002/prot.20375 15645415

[B22] Parthasarathy S Murthy MR Analysis of temperature factor distribution in high-resolution protein structures Protein Sci 1997 6 12 2561 2567 9416605 10.1002/pro.5560061208 PMC2143610

[B23] Yuan Z Zhao J Wang ZX Flexibility analysis of enzyme active sites by crystallographic temperature factors Protein Eng 2003 16 2 109 114 10.1093/proeng/gzg014 12676979

[B24] Hubbard SJ Thornton JM 'NACCESS', computer program Tech. rep., Department of Biochemistry Molecular Biology, University College London 1993

[B25] Kirkpatrick DG Radke JD A framework for computational morphology Computational Geometry, Machine Intelligence and Pattern Recognition 1985 2 217 248

[B26] Liu Q Hoi SC Kwoh CK Wong L Li J Integrating water exclusion theory into beta contacts to predict binding free energy changes and binding hot spots BMC Bioinformatics 2014 15 57 10.1186/1471-2105-15-57 24568581 PMC3941611

[B27] Liu Q Kwoh CK Li J Binding affinity prediction for protein-ligand complexes based on *β *contacts and B factor Journal of Chemical Information and Modeling 2013 53 11 3076 3085 10.1021/ci400450h 24191692

[B28] Martin J Benchmarking protein-protein interface predictions: Why you should care about protein size Proteins: Structure, Function, and Bioinformatics 2014 82 7 1442 1452 10.1002/prot.24512 24420747

[B29] Henrick K Thornton JM PQS: a protein quaternary structure file server Trends in Biochemical Sciences 1998 23 9 358 361 10.1016/S0968-0004(98)01253-5 9787643

[B30] Tarshis L Proteau PJ Kellogg BA Sacchettini JC Poulter C Regulation of product chain length by isoprenyl diphosphate synthases Proceedings of the National Academy of Sciences 1996 93 26 15018 15023 10.1073/pnas.93.26.15018 PMC26348 8986756

